# Recent advances in the biosynthesis and production optimization of gentamicin: A critical review

**DOI:** 10.1016/j.synbio.2024.11.003

**Published:** 2024-11-14

**Authors:** Feng Xu, Kaihao Hu, Ali Mohsin, Jie Wu, Lihuan Su, Yuan Wang, Rong Ben, Hao Gao, Xiwei Tian, Ju Chu

**Affiliations:** Qingdao Innovation Institute of East China University of Science and Technology, State Key Laboratory of Bioreactor Engineering, East China University of Science and Technology, 130 Meilong Road, Shanghai, 200237, People's Republic of China

**Keywords:** *Micromonospora*, Gentamicin biosynthesis, Fermentation process optimization, Molecular regulation, Aminoglycosides

## Abstract

Gentamicin, an aminoglycoside antibiotic, is generated by a few species within the genus *Micromonospora* and has garnered significant attention due to its broad-spectrum efficacy in combating numerous infectious diseases. Comprising a complex array of closely related aminoglycoside compounds, the gentamicin B and C complexes emerge as particularly pertinent in clinical contexts. This review outlines the latest advancements in the biosynthesis and production of gentamicin, commencing with a comprehensive overview of its biosynthetic pathway. Subsequently, the article encapsulates a spectrum of strategies currently deployed to augment gentamicin yields. These strategies include mutation screening, molecular biological techniques, and optimization of the fermentation process. Moreover, numerous methods have been documented for detecting gentamicin across a range of matrices, underscoring the significance of precise quantitative analysis. Finally, the review furnishes an exhaustive market analysis and future outlook, elucidating prevailing trends and challenges within the gentamicin industry. Overall, this article serves as a pivotal resource for researchers and professionals engaged in gentamicin research, furnishing a meticulous introduction to efficient synthesis technologies and diverse applications, alongside presenting innovative concepts and methodologies aimed at increasing gentamicin production.

## 1 Introduction

Antibiotics are a class of secondary metabolites produced by microorganisms, including bacteria, fungi, and actinomycetes, as well as higher plants and animals during their life processes. These substances possess anti-pathogen or other activities and can interfere with the developmental functions of different cells. As the global economy gradually recovers in the post COVID-19 pandemic era, the total population and aging of society increase, and the global pharmaceutical market maintains steady growth. According to data from Grand View Research, the global antibiotic market reached $48.7 billion in 2022 and is projected to reach $68 billion by 2030, with a compound annual growth rate (CAGR) of 4.26 % during the period from 2022 to 2030 (https://www.grandviewresearch.com/). Market Research data indicates that the global antibiotic market was valued at $40.7 billion in 2020 and is anticipated to reach $55.4 billion by 2027, with a CAGR of 4.5 % (https://www.marketresearch.com/). A wide variety of antibiotics exist, among which aminoglycosides are characterized by a core of amino-cyclitol, along with amino sugar rings and glycosidic bonds. These compounds gained prominence as highly effective agents in the treatment of pulmonary tuberculosis due to their potent activity against *Mycobacterium tuberculosis*. Subsequently, the discovery, isolation, and identification of aminoglycoside antibiotics entered a golden age, leading to the discovery and clinical application of numerous additional members of this class. Aminoglycoside antibiotics have been instrumental in the treatment of a wide range of bacterial infections, particularly in combating tuberculosis. In 1944, Selman Waksman identified streptomycin, the first aminoglycoside antibiotic, which was also the first effective therapeutic agent for tuberculosis [1]. This breakthrough not only transformed the approach to tuberculosis treatment but also facilitated the development of subsequent aminoglycoside antibiotics, such as gentamicin (GM), which is a notable aminoglycoside antibiotic that plays a significant role in clinical practice. Due to its broad-spectrum antibacterial activity, rapid bactericidal effect, and low cost, GM has been widely employed in clinical settings. Moreover, GM was once the preferred treatment for Gram-negative bacterial infections. Recently, there has been a gradual increase in international demand for feed-grade GM. According to reports from commercial consulting firms, the global market capacity for GM is expected to grow at a compound annual growth rate of 5.27 % during the forecast period, reaching $688 million by 2028. Thus, the projection indicates that GM will continue to hold a significant share of the aminoglycoside antibiotic market (https://www.globalmarketmonitor.com.cn/index.html).

**Fig. 1:**
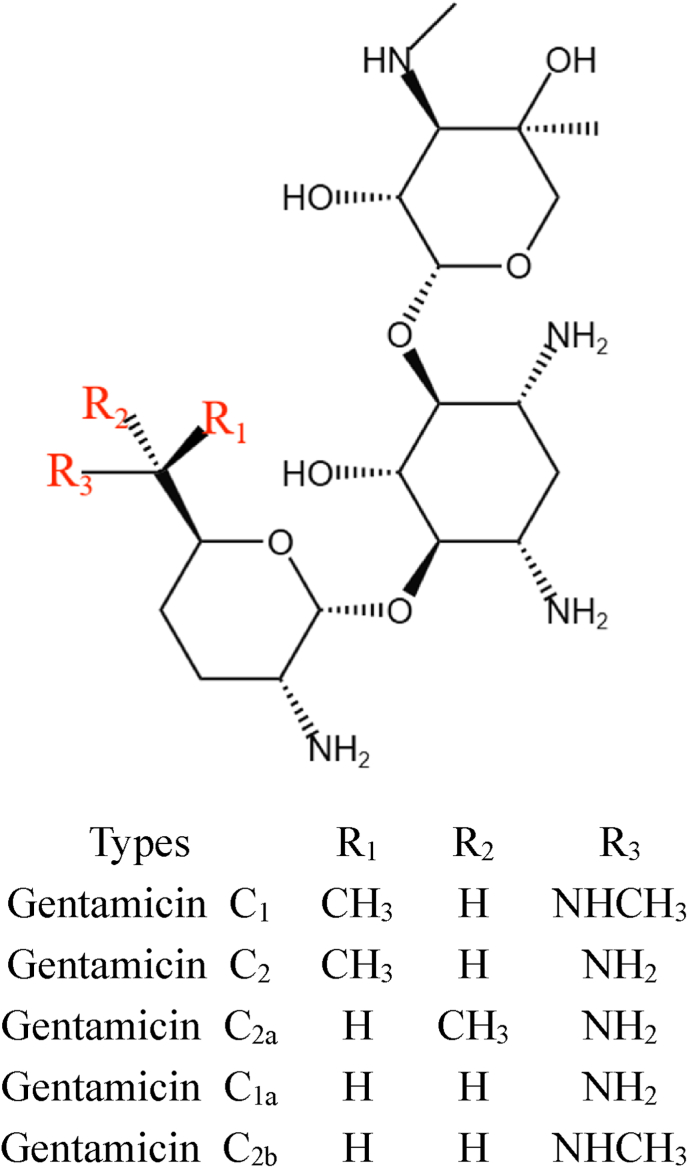
The chemical structure of various gentamicin C complexes.

GM was initially isolated by Weinstein et al., in 1963 from *Micromonospora purpurea* (*M. purpurea*) and *Micromonospora echinospora* (*M. echinospora*), and it was introduced for clinical use in the United States in 1969 [2]. Subsequently, Okachi et al. isolated GM from *Micromonospora sagamienisis* in 1974 and from *M. purpurea* var. *violaceae* from Russia in 1990 [3,4]. GM is a complex mixture composed of 2-deoxystreptamine (2-DOS) as its core, connected to purpurosamine and garosamine via glycosidic bonds [5]. Based on structural variances primarily in the purpurosamine moiety, GM is classified into the A, B, C, X, and sisomicin groups [6]. In clinical practice, the C components of GM are predominantly utilized, comprising C1, C1a, C2, C2a, and C2b (Fig. 1). The primary structural differences among the various GM components are attributed to the linkage patterns and substituents of their sugar moieties. For example, components C1 and C1a differ in the methyl substitution at position 6′, which can influence the drug's stability and solubility [7]. The distinct GM components also exhibit variable antibacterial activity, with some being more efficacious against specific bacterial strains. Notably, C1 and C1a are generally regarded as possessing higher antibacterial potency, whereas C2 and C2a are typically less active [8]. The different GM components may induce varying degrees of adverse reactions. Known side effects of GM include ototoxicity and nephrotoxicity, with certain components more likely to cause these toxicities. For example, components C1 and C1a are reported to have lower toxicity compared to C2 and C2a. In addition, the GM C1a is frequently used as a precursor for synthesizing etimicin, exhibiting the highest potency and the lowest side effects. GM exerts its bactericidal effect by binding to the acyl site on the 16S rRNA of the 30S subunit of bacterial ribosomes within target cells, causing mRNA misreading and disrupting protein synthesis, ultimately leading to pathogen cell death [9]. However, prolonged and extensive clinical usage of GM has inevitably led to significant resistance issues. The widespread occurrence of ototoxicity and nephrotoxicity, typical side effects of aminoglycoside antibiotics, has also constrained the utility of GM [10]. Specifically, aminoglycoside-induced ototoxicity targets the cochlear and vestibular hair cells within the inner ear, resulting in hearing loss or balance disturbances [11]. Furthermore, nephrotoxicity, or kidney injury, is a common adverse effect associated with aminoglycosides. These drugs accumulate in the renal cortex, leading to tubular cell degeneration and ultimately causing acute tubular necrosis [12]. In addition, the emergence of broad-spectrum antibiotics such as β-lactams, quinolones, and cephalosporins, with fewer side effects, has led to a gradual decline in the clinical application of GM. However, the synergistic use of aminoglycoside antibiotics with β-lactam antibiotics has become one of the crucial strategies for combating multidrug-resistant bacteria [13]. Notably, novel biological activities of aminoglycoside antibiotics continue to emerge as an in-depth study. For instance, in antiviral aspects, GM can inhibit the binding of HIV-related peptides to *trans*-activation response (TAR) RNA [14]. Moreover, GM and its metabolic intermediate G418 could restore premature termination codon-induced translation termination, activating the tumor suppressor factor p53 in cancer cells, and leading to apoptosis [15,16]. The novel discovery endows conventional medication with significant potential in cancer treatment. In addition, GM can serve as a sensitizer, significantly enhancing the inhibition and eradication activity against the lung cancer cell line NCI–H460 when used in combination with camptothecin, digoxigenin, and colchicine, among others, in vitro [17]. Encouragingly, the U.S. FDA has approved plazomicin (marketed as ZEMDRI™) developed by Achaogen, chemically modified from sisomicin. Plazomicin can overcome the modification of antibiotic molecules by various aminoglycoside-modifying enzymes, representing the latest generation of aminoglycoside antibiotics targeting multidrug-resistant Gram-negative bacterial infections [18]. Tian et al. employed a simple, low-cost, and scalable one-pot synthesis method at room temperature to fabricate multifunctional TA-Zn-Gen nanoparticles, comprising tannic acid, Zn^2+^, and GM, to treat sepsis [19]. GM and its derivatives are widely utilized as antimicrobial agents in human therapeutics, as veterinary drugs in animal husbandry, and as crop protectants in agriculture [20]. In particular, apramycin is commonly administered in livestock, particularly for the treatment of intestinal infections in pigs, calves, and poultry. Due to its lower toxicity compared to other aminoglycoside antibiotics, apramycin has become a preferred option for veterinary applications [21]. These revelations of novel applications underscore the enduring efficacy and versatility of GM, once a celebrated classic medication, demonstrating its continued relevance and innovation.

This review focuses on the latest advancements in the biosynthesis and production of GM. Initially, the metabolic pathways of GM biosynthesis are introduced. Subsequently, various strategies aimed at enhancing GM productivity are summarized from three perspectives, including mutation screening, molecular biology, and optimization of fermentation processes. Furthermore, an in-depth discussion on methods for determining GM concentration underscores the importance of precise quantification. The comprehensive market analysis further enriches the review by contextualizing scientific advancements within the broader economic landscape, offering strategic insights into market trends and challenges. In conclusion, this review serves as a valuable resource for researchers engaged in GM-related studies, highlighting efficient synthesis processes and diverse applications of GM, while also offering novel insights and methodologies for increasing GM yield.

## 2 The biosynthetic pathway of GM

### 2.1 Identification of intermediate metabolites based on traditional random mutagenesis

GM, a key aminoglycoside antibiotic, occupies an important role in antibiotic research due to its biosynthetic pathway and related metabolites. Investigations of some *Micromonospora* species and their mutants have progressively unveiled the intricate structure and biosynthesis of GM and its derivatives. Weinstein et al. discovered that fermentation products of GM-producing strain contain significant components such as GM C1, C1a, C2, and C2a, and trace compounds with antibacterial activity [22,23]. With advancements in product isolation and analytical detection techniques, a series of compounds including GM C2b and synthetic intermediates A2, X2, G418, JI-20A, and JI-20B have been successively isolated and structurally identified [[24], [25], [26], [27], [28]]. Specifically, *M. purpurea* NRRL 2953 was identified as producing three primary GM components, designated GM C1, C1a, and C2. These components were isolated from the fermentation broth using paper and thin-layer chromatography, employing a mixed solvent system of chloroform, methanol, and 17 % ammonium hydroxide (2:1:1, v/v) [24,25]. These components act as key intermediates, laying the foundation for the synthesis of more complex GM structures. In a separate study, researchers performed an in-depth analysis of the fermentation broth from GM-producing strains, identifying up to 25 aminoglycoside antibiotics. C1, C1a, and C2 remained the primary components, while additional aminoglycosides, including GM A, B, B1, X2, and sisomicin, were identified using mass spectrometry and nuclear magnetic resonance. These results underscore the complexity of the GM biosynthetic pathway and suggest the emergence of various structural variants and degradation products during microbial fermentation [26]. Furthermore, studies of the *M. purpurea* mutant strain JI-33 revealed the production of a novel aminoglycoside, GM C2b. It is a major constituent of the GM C1a complex and corresponds to a minor compound previously detected in the parent strain. This discovery deepens the understanding of key intermediates in GM biosynthesis and suggests that mutations may induce new metabolic branches or promote the synthesis of rare compounds [27]. In addition to GM, the *Micromonospora* species could produce structurally similar aminoglycoside antibiotics. For example, *Micromonospora rhodorangea* NRRL 5326 produces the antibiotic G-418, which displays broad-spectrum antibacterial activity. During its biosynthesis, G-418 incorporates a 2-deoxystreptamine moiety, and after a series of purification steps, including separation via IRA 401S resin and Dowex (1 x 2) resin columns, the free base form of G-418 is obtained. The result underscores the diversity of aminoglycoside biosynthesis in *Micromonospora* strains and suggests the possibility of overlapping or parallel pathways with GM biosynthesis [28]. Identifying these components lays the foundation for elucidating the intricate pathways involved in GM biosynthesis. Testa et al. proposed, for the first time, a model for the biosynthesis of GM C components via two distinct pathways, based on intermediate feeding and bioconversion experiments conducted on GM biosynthesis-blocking mutants obtained through random mutagenesis using conventional physicochemical methods [29]. Subsequently, this research group further elucidated potential steps in GM biosynthesis, including methylation, amination, dehydrogenation reactions, and glycosylation transfers [30]. In summary, beginning with the primary GM components C1, C1a, and C2, it is clear that the biosynthetic pathways for aminoglycoside antibiotics exhibit considerable complexity and diversity across different strains and their mutants. The above studies not only elucidate the core metabolic pathway of GM but also demonstrate structural variations in related aminoglycosides and the discovery of new compounds through the isolation and identification of metabolites during fermentation. However, progress in understanding the GM biosynthetic pathway remained stagnant significantly, primarily due to limitations in molecular genetic manipulation techniques for GM-producing strains.

### 2.2 Resolving biosynthetic pathways based on modern molecular genetics and biochemistry

**Fig. 2:**
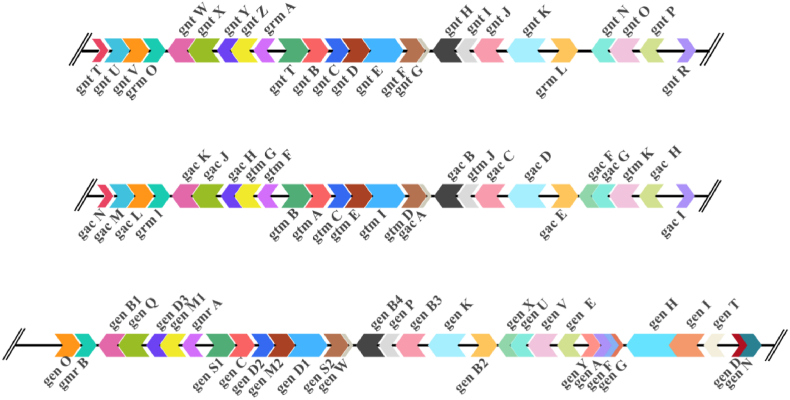
The whole gene sequencing information of gentamicin-producing strains in NCBI (GenBank Accession No. AY524043, AJ575934, AJ628149, respectively).

With the advancement of modern molecular biology and DNA sequencing technologies, elucidating the biosynthetic pathways of aminoglycoside antibiotics such as GM has entered a new phase at the molecular level. Kelemen et al. cloned the *grm*A gene from the GM-producing strain for the first time and demonstrated its role as a GM resistance gene [31]. Subsequently, Wellington et al. utilized conserved sequences within the 2-deoxystreptamine biosynthetic genes as probes to clone the GM biosynthetic gene clusters and completed their sequencing (GenBank Accession: AY524043, AJ575934, AJ628149) [[32], [33], [34]], with each gene cluster being assigned specific names related to GM biosynthesis (Fig. 2). Kharel et al. revealed, through in vitro protein catalysis experiments, that *gtm*A (also known as *gnt*B or *gen*C) is the critical gene responsible for the initial step of GM biosynthesis, converting glucose-6-phosphate to 2-deoxy-scyllo-inosose [33]. This marked the first application of in vitro biochemical enzymology methods in unraveling the mechanism of GM biosynthesis. Furthermore, Park et al. achieved heterologous expression of the nine genes involved in glucose-6-phosphate to GM A2 biosynthesis, initially dispersed within the gene cluster, by recombining them under the erythromycin constitutive promoter P*erm*E∗ and expressed them in *Streptomyces venezuelae* YJ003, successfully detecting the target product GM A2. The study, for the first time from a molecular genetics perspective, confirmed the biosynthetic pathway of GM from the initial synthesis material glucose-6-phosphate to the first trisaccharide intermediate, GM A2 [35].

Due to longstanding difficulties in establishing molecular genetic manipulation systems for GM-producing strains, research on the *in vivo* mechanism of GM biosynthesis has remained stagnant. It was not until 2004 that Wellington confirmed the cloned biosynthesis gene cluster (BGC) of GM using homologous recombination-based gene insertion inactivation methods [32]. Subsequently, Kwon et al. replaced the methyltransferase homolog gene *gnt*E (also known as *gtm*I or *gen*D1) in the BGC of GM with a streptomycin resistance gene, leading to the accumulation of the intermediate product GM A2 in mutant strains. The study proposed that *gnt*E is responsible for the initial modification of GM A2, a pseudotrisaccharide intermediate, in GM biosynthesis [36]. In addition, Hong et al. employed gene replacement techniques to replace the erythromycin resistance gene with another methyltransferase homolog gene, *gnt*K (also known as *gac*D or *gen*K), within the BGC. This resulted in mutant strains no longer producing GM C components (C1 and C2) with methylation modification at C-6′, while significantly increasing the yield of GM C component (C1a) without methylation modification at C-6'. This highlights the role of *gnt*K in C-6′ methylation modification [37]. Xia et al. obtained high-yield mutant strains of GM C1a by *in vivo* knockout of *gac*D (also known as *gnt*K or *gen*K) [38]. Liu et al. successfully expressed the cobalamin-dependent methyltransferase *gen*K in vitro and conducted protein catalysis experiments using GM X2 as a substrate. The production of methylated product G418 at C-6′ was observed, providing biochemical enzymology evidence that *gen*K encodes the catalysis of GM C-6′ methyl transfer [39]. In addition, Chen et al. conducted catalytic reactions using in vitro expressed *gnt*I (also known as *gtm*J or *gen*P) with kanamycin B (similar to GM JI-20A), successfully detecting kanamycin B with phosphorylation modification at C-3'. It was inferred that *gnt*I also catalyzes the phosphoric acid transfer at C-3′ of GM, serving as a prerequisite for the hydroxy group removal in JI-20A and JI-20B [40]. These findings represent a deeper understanding of the biosynthetic pathway of GM, elucidating the functions of individual genes within the BGC and paving the way for a comprehensive molecular-level explanation of GM biosynthesis.

In 2014, Sun et al. comprehensively utilized molecular genetics and biochemical methods to systematically elucidate the biotransformation process from the initial trisaccharide compound GM A2 to the pivotal branching point GM X2 [41]. They employed in-frame deletion to inactivate four critical genes involved in this pathway: *gen*D2 (also known as *gnt*C or *gtm*C), *gen*S2 (also known as *gnt*F or *gtm*D), *gen*N, and *gen*D1. Subsequently, they fed intermediate products to the mutant strains and predicted the corresponding relationship between the deleted target genes and catalytic steps based on changes in fermentation products. Specifically, *gen*D encodes the dehydrogenase catalyzing the oxidation of the hydroxyl group at C-3″ of galactosamine, *gen*S2 encodes the aminotransferase responsible for the formation of the amino group at C-3″ of galactosamine, *gen*N encodes the methyltransferase catalyzing the methylation of the amino group at C-3″ of galactosamine, and *gen*D1 encodes the methyltransferase catalyzing the methylation at C-4″ of galactosamine. Meanwhile, they confirmed that *gen*K encodes the methyltransferase responsible for catalyzing the formation of GM A2e from GM A2, and accumulation of GM Ae was detected in *Δgen*D1 mutants. In addition, GM A2 was isolated from *Δgen*S2*Δgen*K mutants and utilized as a substrate in enzymatic catalysis reactions with GenD2, GenS2, and GenN expressed in vitro, leading to the production of GM A. Subsequent catalysis by GenD1 resulted in the conversion of GM A to GM X2, thus reconstructing the biosynthetic pathway in vitro [41]. GM X2 is a central intermediate in GM biosynthesis and a crucial branching point leading to the formation of multiple components of GM C. Despite its chemically simple aminoglycoside structure, GM exhibits surprisingly diverse methylations. These methylations serve as pivotal functional groups showcasing GM's structural diversity and biological activity while constituting critical structural units involved in resistance development.

Subsequently, *gen*Q and *gen*K were individually and concurrently deactivated by in-frame deletion. Combining feed experiments on mutant strains, it was elucidated that *gen*Q governs the pathway of GM C components devoid of C-6′ methylation modification, while *gen*K is responsible for the pathway of GM C components with C-6′ methylation modification. GenK plays a pivotal role in GM biosynthesis, particularly at a key branching point within the pathway. It functions as a cobalamin (vitamin B12)-dependent radical S-adenosyl-l-methionine (SAM) methyltransferase, catalyzing the methylation of the 6′-carbon of GM X2 (GenX2) to produce the critical intermediate G418 [42]. This methylation event constitutes a major branching point in the GM biosynthetic route, determining the direction of the metabolic flux. Specifically, GenK-mediated methylation guides the pathway toward the biosynthesis of GM C2, C2a, and C1, whereas the absence of this methylation results in the formation of GM C1a and C2b. The identification of this branching point provides vital insights into the regulation of the GM biosynthetic pathway [43]. Furthermore, in vitro studies of GenK activity have elucidated its catalytic mechanism, demonstrating the transfer of the S-methyl group from SAM to cobalamin and aminoglycoside substrates, accompanied by the production of 5′-deoxyadenosine and S-adenosylhomocysteine [39].

In addition, single-gene and multigene deactivation were performed on four genes encoding pyridoxal phosphate (PLP)-dependent aminotransferases: *gen*B1 (also known as *gnt*W or *gac*K), *gen*B2 (also known as *gnt*L or *gac*E), *gen*B3 (also known as *gnt*J or *gac*C), and *gen*B4 (also known as *gnt*H or *gac*B), followed by the expression of the proteins encoded by these genes in vitro. While all these enzymes rely on PLP, they exhibit distinct catalytic reactions and evolutionary relationships. GenB1 and GenB2 primarily mediate the transamination of GM X2, converting it to JI-20A and JI-20Ba, which are precursors to GM C1a, C2a, and C2 [44]. GenB3 is implicated in the dideoxygenation modification within GM biosynthesis, a complex process involving sequential dehydroxylation and reduction of double bonds [45]. GenB4, a bifunctional enzyme with both reductase and transaminase activities, catalyzes the final step in the 3′,4′-dideoxygenation modification of GM [46]. Evolutionarily, GenB1, GenB2, GenB3, and GenB4 are likely derived from a common ancestral gene. They have evolved into specialized enzymes with distinct functions through gene duplication and subsequent divergence. This evolutionary divergence may involve key amino acid residues alterations, thereby influencing their catalytic properties and substrate specificities. Specifically, through joint catalysis with GenQ, it was observed that the proteins encoded by the four genes could catalyze the amino transfer process of GM C-6′ to varying degrees, with GenB1 exhibiting the highest catalytic efficiency. Furthermore, it was discovered that GenB2 possesses isomerization activity for GM C2a and C2, while GenB3 and GenB4 likely participate in the deoxygenation process at C-3′ and C-4′ of GM C [43]. This research, focused on the pivotal branching point GM X2 in the GM biosynthetic pathway, comprehensively elucidated the synthesis origin of GM X2 and the route leading to downstream products. It provides a theoretical basis for the efficient and targeted optimization and innovation of GM through combinatorial biosynthesis methods and technologies. Gu et al. disrupted *gen*B1 and *gen*B2 in BGC, resulting in high-yield strains producing intermediate metabolites like G418, thus confirming the existence of isomerization reactions in GM biosynthesis [47]. Using GM X2 and G418 as substrates, one pathway biosynthesizes GM C1a and GM C2b with a 6′-nonchiral carbon, while another pathway produces GM C2, C2a, and C1 with a 6′-chiral carbon. When GM X2 serves as the substrate, GenQ and GenB1 catalyze the dehydrogenation/transamination of GM X2 to produce JI-20A, which, after 3′,4′-deoxylation, forms GM C1a. Likewise, GenQ and GenB1 catalyze the dehydrogenation and transamination of G418 to generate JI-20Ba with a 6′(S)-configuration, which, after 3′,4′-deoxylation, forms GM C2a. GenQ and GenB2 catalyze the dehydrogenation and transamination of G418, forming JI-20B with a 6'-(R)-configuration. Subsequent 3′,4′-deoxylation of JI-20B produces GM C2. Moreover, GenB2 acts as a bifunctional enzyme with isomerase activity, catalyzing the isomerization reactions of JI-20Ba (JI-20B) and GM C2a (GM C2) [45]. During the processes where JI-20A and JI-20B are respectively deoxygenated to GM C1a and GM C2, the 3′-OH phosphorylation catalyzed by the phosphotransferase GenP is confirmed as the first step of deoxygenation. The 5′-pyridoxal phosphate (PLP)-dependent enzyme GenB3 further utilizes these phosphorylated substrates for deoxygenation, forming the 3′,4′-deoxy-4′,5′-ene-6′-oxides. Subsequently, GM C components are formed through C-6′-transamination and 4′,5′-ene reduction catalyzed by genB4 [46,48]. In conclusion, GenB1, GenB2, GenB3, and GenB4 perform distinct roles in the GM biosynthetic pathway. Their evolutionary relationships and functional diversification provide diverse catalytic mechanisms, which are essential for the biosynthesis of GM. In addition, recent advancements in structural biology have greatly improved our understanding of key enzymes in the GM biosynthetic pathway. The Dias research group elucidated the crystal structure of the methyltransferase GenN, confirming its methylation activity across a range of substrates [49]. They identified the structural basis for its broad substrate specificity by analyzing its three-dimensional structure. The group also determined the structure of another key enzyme in GM biosynthesis, GenD2, an NAD^+^/NADP^+^-dependent oxidoreductase involved in dehydrogenating the C-3″ hydroxyl group. Structural analysis revealed two distinct β-folds and examined their influence on dimer formation and substrate recognition [50]. In addition, GenB2, an enzyme in the GM biosynthetic pathway, catalyzes the epimerization at the C-6′ position of garosamine. Resolving the crystal structure of GenB2 in complex with substrate analogs enabled researchers to determine its catalytic mechanism and verify the role of critical amino acid residues via site-directed mutagenesis [44]. Studies on GenB3 and GenB4 revealed that despite their highly similar amino acid sequences, they exhibit distinct functional differences. Serine at position 57 in GenB3 and aspartic acid at position 52 in GenB4 were identified as key determinants of their functional differences. A single amino acid substitution leads to substantial functional divergence between these two enzymes [45]. These structural biology findings advance our understanding of GM biosynthesis and provide critical theoretical insights for enzyme engineering, potentially enhancing GM yield or improving its pharmacological properties.

**Fig. 3:**
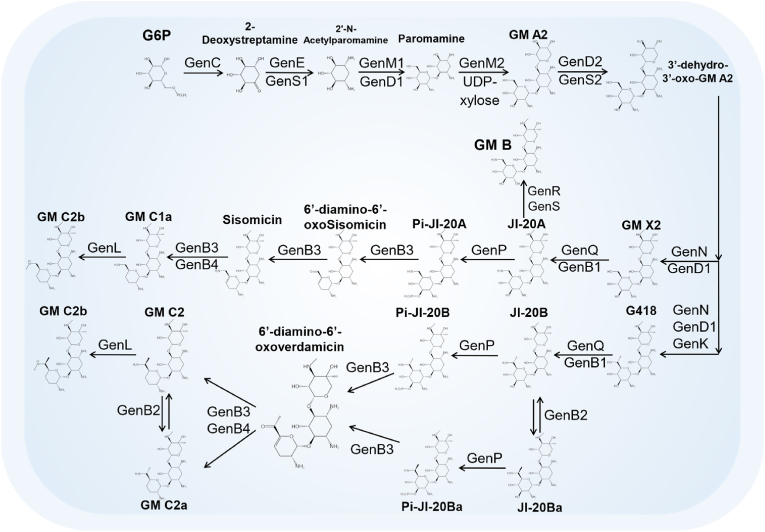
The known GM biosynthesis pathway by *M. echinospora.*

In the biosynthetic pathway of GM, N-6′ methylation is crucial among all methylation modifications. This modification plays a key role in metabolically directed blocking pathways to obtain the GM C1a, and the low-toxicity single component C2. However, its encoding gene has remained elusive despite exhaustive examination through gene knockout of all potential methylation genes in the BGC of GM. Ultimately, Sun et al., through whole-genome sequencing and a combination of in vitro enzymology and *in vivo* genetics engineering, successfully located the last methylation gene, *gen*L, responsible for the final step of GM biosynthesis [42]. Surprisingly, the gene was 2.54 Mb away from the GM biosynthetic gene cluster on the chromosome, filling the last gap in the GM methylation modification network. Moreover, the study revealed that the methylation catalyzed by the methyltransferases GenN, GenD1, and GenK at the C-6′, N-3″, and C-4″ positions, respectively, is relatively independent. This breakthrough challenges the traditional notion of a "serial" methylation sequence in GM and establishes, for the first time, a "parallel" multistep methylation modification stereo network model [42]. Overall, the biosynthetic pathway of GM C components has been elucidated (Fig. 3).

The biosynthetic pathway of GM C components is well-documented, but there is limited literature on GM B synthesis pathways. Additionally, since GM B cannot convert into any GM C components, it is not considered an intermediate in the GM C component biosynthesis pathway [51]. Ban et al. demonstrated through in vitro experiments that GM B components can be assembled via three independent biosynthetic pathways, revealing seven newly identified intermediates [52]. The elucidation provides detailed genetic and molecular insights for enhancing GM B production. Furthermore, Chang et al. employed *kan*J and *kan*K as probes to conduct genomic mining of the GM B-producing strain *M. echinospora* CCTCC M 2018898, identifying potential deamination-related genes, *gen*R, and *gen*S. Validation via *in vivo* knockout and complementation experiments confirmed the involvement of *gen*R and *gen*S in the final step of GM B biosynthesis [53].

The studies summarized here represent significant progress in understanding the molecular details of GM biosynthesis, driven by advances in molecular biology, genetics, and biochemical techniques. The discovery of essential genes, such as *grm*A for GM resistance and *gtm*A for the initial biosynthetic step, laid the groundwork for subsequent research. The heterologous expression of crucial genes in *Streptomyces venezuelae* further confirmed the pathway from glucose-6-phosphate to GM A2, while gene disruption experiments have elucidated the roles of methyltransferases such as *gnt*E, *gnt*K, and *gen*K in the modification of GM intermediates. Moreover, in vitro enzymology experiments have proven invaluable in confirming these roles, particularly in the methylation modifications that are critical for the structural diversity and biological activity of GM. Notably, GenK functions as a critical branching point in the biosynthetic pathway, mediating a methylation reaction that directs one metabolic branch toward the formation of GM C2, C2a, and C1, while the unmethylated pathway leads to the production of GM C1a and C2b. In addition, the discovery of the *gen*L gene, encoding the final step of methylation in GM biosynthesis, marks a significant breakthrough, challenging the previous understanding of the methylation process and revealing a more complex, "parallel" multistep modification network. Finally, the research into GM B biosynthesis pathways has expanded the understanding of GM production, distinguishing the independent biosynthetic routes for GM B and uncovering novel intermediates. These studies advance the fundamental knowledge of GM biosynthesis and open up potential biotechnological applications, such as the targeted engineering of GM components to improve yield and reduce toxicity. In addition, the enhancement is crucial for advancing fermentation processes, which will be explored in the subsequent section.

## 3 Strategies for strain performance improvement

### 3.1 High-throughput mutagenesis screening for high-yielding strains

Microbial strain improvement, as a pivotal component of fermentation processes, significantly impacts the enhancement of GM production. Classical strain improvement techniques such as physical or chemical mutagenesis are employed to select forward mutants that elevate metabolite yields or mitigate metabolite deactivation. For GM production, Yang et al. conducted ultraviolet mutagenesis on the original strain and identified high-yield strains, which exhibited a 34.3 % titer increase in shake flasks compared to the parental strain [54]. Zhao et al. utilized N^+^ ion beam irradiation for strain mutagenesis, resulting in a 27.39 % yield enhancement compared to the wild-type strain [55]. In addition, Wu et al. employed a composite mutagenesis approach using N^+^ ion beam irradiation and ultraviolet radiation on *M. purpurea*, followed by high concentrations of GM as selection criteria, culminating in a 75.2 % titer increase in shake flasks compared to the parental strain [56]. Moreover, Ni et al. utilized N-methyl-N′-nitro-N-nitrosoguanidine (NTG)-induced random mutagenesis to augment G418 production, achieving a 26.6 % further yield enhancement through two successive random mutagenesis steps [57]. In recent years, atmospheric room-temperature plasma (ARTP) technology has found wide applications in mutation breeding. This technique, based on the principle of atmospheric pressure radio frequency glow discharge, generates a high-concentration stream of active particle plasma at temperatures ranging from 25 to 40 °C, capable of penetrating cells and inducing DNA mutations [58]. ARTP mutagenesis offers significant advantages, including operation at room temperature, simplicity, rapid processing, high safety, and low cost [59]. Tian et al. investigated various mutagenesis methods on *M. purpurea*, obtaining stable high-yield strains through high-throughput screening. Ultimately, in a 5 L fermentation tank validation experiment, the AL324 strain exhibited an 81.3 % titer increase compared to the parental strain [60]. Zhu et al. employed ARTP mutagenesis on an industrial GM-producing strain and successfully screened one genetically stable high-yielding strain (998 U/mL) out of 3005 mutants. Validation experiments demonstrated a 72.7 % titer increase in the 5 L bioreactor [61]. Ge et al. subjected the parental strain *M. purpurea* GM20190109-15 to ARTP and lithium chloride composite mutagenesis. Through high-throughput screening, a mutant strain, CH20190225-107, was obtained, showing a titer increase from 1547 U/mL to 2280 U/mL, with a 47.4 % titer increase in shake flasks compared to the parental strain [62] (Table 1). Classical strain improvement techniques have played a crucial role in enhancing GM production. However, the time-consuming and labor-intensive nature of these methods highlights the need for more efficient approaches. This sets the stage for discussing how metabolic engineering strategies can offer a more targeted and effective solution, as explored in the next section.

**Table 1:** The different mutagenesis methods and results in producers of GM**.**

Parent strain	Breeding method	GM yield after mutagenesis	Determination methods	Increase in fermentation yield	References
*M. purpurea* JY1-12	UV	2078 U/mL	Microbiological assay	34.3 %	[54]
*M. echinospora*	N^+^ implantation	1800 U/mL	Microbiological assay	27.39 %	[55]
*M. purpurea*	N^+^ implantation; UV	2118 U/mL	Microbiological assay	75.2 %	[56]
*M. echinospora* SPU336	NTG-induced stochastic mutagenesis	861 U/mL	High-performance liquid chromatography - evaporative light scattering detector (HPLC-ELSD)	26.6 %	[57]
*M*. *purpurea*	ARTP; LiCl	1193 U/mL	Ultraviolet spectrophotometry	81.3 %	[60]
*M. echinospora* 49-92S	ARTP	998 U/mL	HPLC-UV	72.7 %	[61]
*M*. *Purpurea* GM20190109-15	ARTP; LiCl	2280 U/mL	HPLC-UV	47.4 %	[62]
*M*. *sagamiensis* ATCC 21826	Femtosecond laser (wavelength of 800 nm, repetition frequency of 76 MHz, pulse time of 150 fs)	126 U/mL (GM C2b)	Microbiological assay	2.15-folds	[63]
*M*. *purpurea* KR 960796	neutron radiation (30 KeV)	–	Microbiological assay	46 %	[64]
*M*. *purpurea* 23–18	^12^C-ion beam irradiation	1306 U/ml	Microbiological assay	20 %	[65]
*M*. *echinospora* var. 23-18	Penicillin and ultrasonic treatment;^60^Co mutagenesis	–	HPLC-UV	20 %	[66]

### 3.2 Metabolic engineering strategies to enhance GM production

**Fig. 4:**
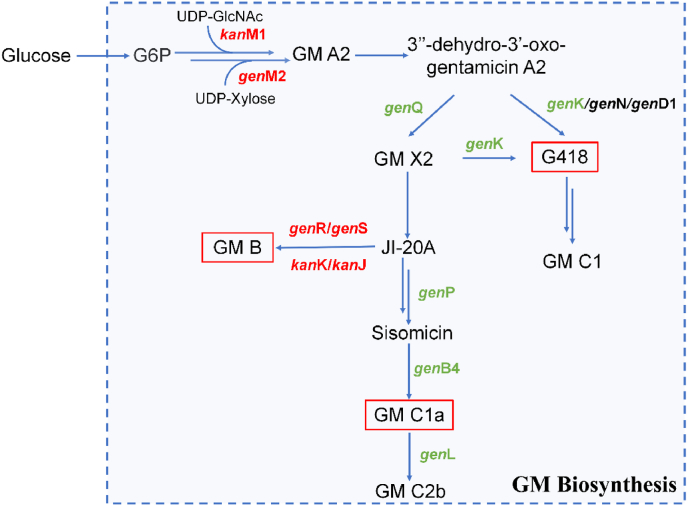
The enhancement strategy of GM production by metabolic engineering. The genes that have been overexpressed are indicated in red, and knockouts are marked in green.

Although traditional random mutagenesis methods can achieve specific results without genomic information or genetic tools, they are often time-consuming and labor-intensive. Therefore, engineering high-yield strains based on metabolic engineering strategies can effectively enhance the production levels of specific components (Fig. 4). Li et al. focused on *M. purpurea* and identified the *gac*D gene responsible for converting GM X2 to G418 through sequence analysis. By inactivating the *gac*D gene, they obtained an engineered strain with a component C1a yield about ten times higher than before [38]. With the recent revelation of the last methyltransferase, *gen*L, involved in GM C2b biosynthesis, Zeng et al. further knocked out both *gen*K and *gen*L in *M. purpurea*. Mass spectrometry analysis confirmed that the engineered strain did not produce GM C2b [67]. Furthermore, Xu et al. also performed simultaneous knockout of *gen*K and *gen*L in the industrial strain *M. echinospora* 49-92S, resulting in an approximately 3.22-fold increase in the C1a component compared to the parental strain [68]. This series of studies has made it possible to obtain single-component GM engineered strains and apply them to industrial production. Rajasekaran et al. synthesized GM B1 and X2 minor components in vitro using precursor substances, paving the way for the exogenous synthesis of GM [69]. Wu et al. constructed a *gen*Q knockout strain, YC005, based on the industrial strain *M. echinospora* J1-020 as the chassis organism. Fermentation results showed that the YC005 strain only produced a single component, G418, at a 460 mg/L yield. Starting from the YC004 strain, *gen*B4 and *gen*K were successively knocked out to generate the double knockout mutant YC007. Shake flask fermentation results demonstrated that the YC007 strain only produced a single component, sisomicin, at a yield of 1046 mg/L [70]. According to the known GM biosynthesis pathway, UDP-N-acetyl-d-glucosamine (UDP-GlcNAc) and UDP-xylose are fundamental building blocks. The genes of *kan*M1 and *gen*M2 are critical regulators in this process. The previous results have demonstrated that overexpression of *kan*M1 and *gen*M2 in *M. echinospora*, coupled with appropriate glucose supplementation during fermentation, can increase production by approximately 45 % [71]. Moreover, providing only precursors, such as adding xylose during GM C1a fermentation, can also enhance production by 31.6 % compared to previous methods [72].

The GM B acts as the primary precursor for isepamicin. Despite being a minor metabolite, molecular techniques can significantly enhance its production. Ni et al. engineered a high-yield strain of JI-20A by deleting the *gen*K and *gen*P genes, crucial for GM B production. They subsequently introduced the *kan*K and *kan*J genes from the kanamycin gene cluster via heterologous expression, substituting a weak promoter with a robust one, resulting in an efficacy of 880 μg/mL for the B component [73]. In addition, Chang et al. employed a high-intensity promoter to successfully develop strains with proficient expression of *gen*R and *gen*S, leading to a GM B yield of 798 mg/L, marking a 64 % increase compared to the original strain [53].

In summary, GM production has progressed significantly through traditional and modern strain improvement techniques. Traditional mutagenesis methods, such as UV radiation, ion beam irradiation, and combined mutagenesis, have consistently generated strains with notable titer increases, ranging from 26.6 % to 81.3 %. The adoption of ARTP mutagenesis technology has recently accelerated these advances, offering a more efficient approach to developing high-yielding strains. However, despite their success, traditional methods are labor-intensive and require high-throughput screening, highlighting their limitations. In contrast, metabolic engineering provides a more targeted and efficient alternative. Gene manipulation studies on *M. purpurea* and *M. echinospora* have enabled precise modifications to biosynthetic pathways, resulting in substantial increases in the production of specific engineered components. These approaches have led to the creation of single-component strains and significant yield improvements, in some cases up to 10-fold. By utilizing genetic tools to fine-tune critical metabolite production, metabolic engineering offers a promising avenue for further optimization. Overall, these studies emphasize the complementarity of traditional and modern techniques in strain improvement. In addition, optimizing fermentation processes is crucial for maximizing GM production yields and efficiency. The next section will explore the interplay between strain improvement and fermentation optimization, highlighting integrated strategies for superior GM production.

## 4 Fermentation process optimization for GM production

### 4.1 Media optimization

The medium supplies vital nutrients for microbial growth and metabolism. Optimizing the nutrient composition in GM fermentation is crucial to creating an environment conducive to the development and metabolism of production strains, thereby enhancing GM yield. Chen et al. conducted orthogonal experiments using *M. purpurea* 2–25 and found soybean meal to have the most significant impact on fermentation. They determined an optimal proportion, resulting in a 35 % reduction in fermentation time and a 43 % increase in GM titer [74]. Meenavilli et al. employed response surface methodology to optimize critical medium components for GM production, identifying soybean meal, cobalt chloride, potassium dihydrogen phosphate, and starch as crucial factors. They concluded that a soybean meal content of around 3.0 % was optimal for GM production [75]. Fang et al. demonstrated that substituting appropriately hydrolyzed starch solution for corn starch in the fermentation medium of *M. purpurea* G-105 increased antibiotic production [76]. Feng et al. found that approximately 3.0 % of soybean meals were most conducive to antibiotic production, with heat-extracted and fresh soybean meals showing the highest effectiveness [77]. Wang et al. investigated the effect of CoCl_2_ addition, revealing that it significantly boosted GM production [78]. Hong et al. optimized the nutrient formula for GM B fermentation using Plackett-Burman experiments, identifying KNO3, soybean meal, and CoCl_2_ as critical factors, resulting in a 20 % increase in yield [79]. Wu et al. explored the role of Fe^3+^ ions in fermentation, noting their inhibitory effect on antibiotic biosynthesis, which could be mitigated by adding water in the final fermentation stage [80]. Abrasimovski et al. studied the impact of orthophosphate on GM-producing strains, revealing that increased inorganic phosphate content promoted strain growth but inhibited antibiotic biosynthesis, with higher activity strains being more sensitive to this effect [4]. However, optimization schemes based on classical Design of Experiments (DOE) remain labor-intensive and time-consuming for enhancing the yields of high-performance strains. The advent of machine learning and genome-scale metabolic network models is increasingly being applied in biotechnology and bioprocess [81,82]. Future efforts to optimize engineered strain cultivation should prioritize selecting and implementing these advanced methods to improve GM production further.

### 4.2 Fermentation process regulation

The addition of specific components during fermentation is essential for optimizing microbial metabolism and enhancing antibiotic production. Common supplements, such as inorganic salts, peptone, and amino acids, boost cellular metabolism, promote growth, and increase yields. Yang et al. explored the impact of organic and inorganic salts on GM production [83]. They discovered that supplementing the culture medium with 0.1 % calcium chloride (CaCl_2_) and 0.3 % sodium citrate increased GM yield by 11.5 %, elevating the titer from 2150 μg/mL to 2398 μg/mL. Furthermore, the proportion of GM C1a rose from 38 % to 42 %. Researchers elucidated the underlying mechanism through untargeted proteomic techniques: calcium chloride addition modified methylation during GM biosynthesis, thereby augmenting C1a production. Meanwhile, sodium citrate inhibited primary cell metabolism, thus fostering the generation of GM as a secondary metabolite. The investigation lays a scientific foundation for producing high-purity GM C1a and various GM components. Liu et al. supplemented the culture with 0.4 % peptone in the late fermentation stage, increasing GM production by 3.23 % and reducing C2 and C2a content [84]. Fan et al. employed intermittent feeding with multiple small carbon source additions to enhance oxygen levels, resulting in an 87.3 % increase in fermentation units and a prolonged 5-day production cycle. As fermentation progresses, strain growth leads to elongated and aggregated mycelia, decreasing dissolved oxygen and significantly hindering antibiotic production [85]. Himabindu et al. explored non-nutritional stress effects, revealing heat shock, high ethanol concentration, and high NaCl concentration as effective in boosting GM production [86]. In addition, Guan et al. added glycine, serine, lysine, and tyrosine during fermentation, enhancing cell metabolism, shortening fermentation time, and increasing yield by 30%–95 % [87]. In conclusion, strategically incorporating these ingredients during fermentation is crucial for optimizing the metabolic environment, improving production efficiency and product quality.

Notably, factors like culture temperature, time, and method are critical fermentation process parameters that directly impact the outcomes of antibiotic fermentation. Yang et al. optimized inoculum age, volume, culture temperature, initial pH, speed, and duration, achieving a GM titer of 1824 U/mL [88]. Feng et al. found that adding sterile water and controlling the loading coefficient to 0.8 in the middle-to-late stages of GM fermentation increased dissolved oxygen, alleviating oxygen deficiency [78]. Filamentous growth of *Streptomyces griseus* during fermentation can be disrupted by excessive agitation, affecting growth and metabolism. Thus, impeller design and stirring speed significantly affect GM production. Guo et al. showed that three-blade six-leaf impellers improve metabolic activity significantly, increasing product levels by approximately 48.6 % compared to two-blade six-flat leaf turbine impellers [89]. In addition, Meenavilli et al. investigated the fermentation parameters of *M. purpurea* in a 5 L stirred fermenter, optimizing the air-to-liquid ratio and speed to achieve a threefold increase in GM concentration compared to shake flasks [75]. Recent advances in artificial intelligence (AI) have propelled the intelligentization of microbial fermentation to the forefront of academic research [[90], [91], [92]]. The data generated by microbial fermentation are rich in dimensions and contain complex intrinsic relationships that can be deciphered using AI-based machine learning techniques. Therefore, soft sensors constructed using various algorithms have emerged as an innovative technology in microbial fermentation research [[93], [94], [95]]. These soft sensors can integrate relevant variables, including substrate concentration, pH, and real-time monitoring parameters, to predict critical parameters in the microbial fermentation process [91,92]. Therefore, AI-driven soft sensing technology not only paves a new path for the intelligentization of microbial fermentation but also holds the potential to significantly enhance the efficiency and quality of this process, further advancing the research and development of the microbial fermentation discipline.

### 4.3 GM secretion and release

The limited membrane permeability, substantial cell wall thickness, and dense peptidoglycan layer of *M. purpurea* result in notable retention of antibiotics within cells during GM fermentation, rather than their release into the extracellular milieu, thereby impacting GM biosynthesis and final production levels. Antibiotics are typically secreted through two primary processes: efflux across the cell membrane into the extracellular space and detachment from the cell wall into the fermentation broth. Augmenting either process enhances secretion, while inhibition reduces secretion rates [96]. Notably, peptidoglycan on the cell wall significantly impedes GM release. Typically, only 20–30 % of GM is secreted into the fermentation broth during fermentation, with the remaining approximately 75 % bound to the cell wall [97]. Reiblein et al. observed that ultrasound could partially disrupt the nonspecific binding between GM and mycelia, although complete GM release was not achieved. Xiong et al. increased GM secretion by modifying cell membrane permeability by adding surfactants and metal ions to the culture medium [98]. Xiong and Zhang et al. investigated GM secretion during fermentation, demonstrating that ultrasound and ion stimulation could enhance GM release [99,100]. Application of ultrasound at approximately 84 h of GM fermentation increased release by 70 %, and offline ultrasound treatment raised intracellular GM release from 38.3 % to 75.8 % [101]. Furthermore, metal ions like Mg^2+^ and Na^+^ during fermentation can promote GM release by competing with GM for binding sites on the cell wall [102]. Guan et al. augmented GM production by 65 % by adding 0.05 % and 0.15 % N-acetylglucosamine to the culture medium at 48h and 60h, respectively, thereby partially or entirely alleviating the GM adsorption in the cell wall [103]. The application of *in situ* separation technology in microbial fermentation aids in increasing product yield and quality, reducing production costs, and enabling the possibility of continuous fermentation on an industrial scale [[104], [105], [106]]. Thus, *in situ* separation technology is expected to demonstrate significant potential and application prospects in promoting microbial fermentation and industrial GM production.

A substantial body of research has outlined various strategies for optimizing nutrient composition, fermentation parameters, and secretion mechanisms to enhance GM production. Studies suggest that an optimal soybean meal concentration of approximately 3 %, combined with careful management of other nutrients such as calcium chloride and starch, creates the most favorable conditions for high GM titers. Moreover, targeted supplementation with calcium chloride and sodium citrate increases GM yields and raises the proportion of specific components, such as C1a, providing a pathway for producing higher-purity antibiotics. Regarding the fermentation process, physical parameters like inoculum age, temperature, dissolved oxygen levels, and agitation have been identified as critical factors for GM production. Techniques such as intermittent carbon feeding and mechanical adjustments, including impeller design, have effectively mitigated oxygen-related bottlenecks and enhanced antibiotic yields. Furthermore, studies on cell wall modulation, notably the addition of N-acetylglucosamine, have shown that weakening the cell wall's binding strength significantly improves GM secretion by reducing intracellular retention, thereby increasing the availability of GM in the fermentation broth. Together, these findings emphasize the crucial role of media optimization and fermentation parameter adjustment in maximizing GM production.

## 5 The determination of GM

The intricate structure and multi-component composition of GM result in the excretion of most of its components unchanged in urine and faeces, contributing to soil and natural water pollution. Hence, accurate determination of GM and its component concentrations is paramount. This section aims to delineate diverse analytical methods employed for the quantitative or qualitative detection of GM and its sulfate in various matrices, with the aspiration of advancing novel developments in GM analysis technology across diverse application domains. Table 2 presents a compendium of quantification methods for GM, encompassing spectrophotometry, fluorescence, liquid chromatography, ultra-high-performance liquid chromatography-tandem mass spectrometry, electrochemistry, colorimetry, chemiluminescence, and capillary electrophoresis.

**Table 2:** The various quantification methods for GM in multiple matrices.

Methods	Advantage	Disadvantage	The situation using the method	The measurement range	Reference
Capillary electrophoresis	Simple operation, no derivatization required	Low measurement accuracy, not sensitive enough to analyze impurities	Suitable for the analysis of GM active ingredient in APIs	Nanogram level (ng)	[[113], [114], [115], [116]]
Fluorescence	High selectivity, high sensitivity, simple instrumentation, good reproducibility	Determination of liposomes must avoid or remove other molecules containing imine groups	For quantification of drugs in liposomes, also other samples containing GM	Nanogram level (ng)	[117,118]
Chemical luminescence	High sensitivity, simple operation, low reagent usage, good reproducibility, low cost	Susceptible to interference by impurities, samples need to be pre-treated	Application to the determination of gentamicin sulfate in preparations and water samples	Microgram level (μg)	[[119], [120], [121], [122]]
Ultraviolet spectrophotometry	Easy and fast to operate, reasonable specificity, good reproducibility	Measurement linear range is limited	Suitable for quality control analyses of GM, especially where fast and easy operation is required	Microgram level (μg)	[95,96]
HPLC-CAD	High sensitivity, easy handling, no derivatization required, high selectivity	Higher equipment costs and more complex data processing	Suitable for stability indication analysis of pharmaceuticals containing gentamicin	Microgram level (μg)	[[123], [124], [125]]
HPLC-ELSD	Simple to use, no need for specific standards, high accuracy	Higher equipment costs and more complex optimization of the detection response of ELSDs	General detection methods for non-absorbing analytes	Microgram level (μg)	[[127], [128], [129], [130], [131]]
chromatography-mass spectrometry	Fast and precise, high specificity, low contamination of the mass spectrometer	Higher equipment costs and higher requirements for technicians	For quality control and impurity analysis of GM in the pharmaceutical industry, pharmacokinetic studies	Nanogram level (ng)	[[132], [133], [134], [135], [136]]
Colorimetric method	Good linear range, low detection limit, short detection time, high accuracy, reasonable specificity	The corresponding metal nanoparticles have a high pH requirement	Qualitative and quantitative analysis of GM in dairy products	Nanogram level (ng)	[108,109,[137], [138], [139], [140]]
Electrochemical method	Low cost, high selectivity, trace detection capability and high analytical frequency	Problems with electrode passivation, sometimes complicated sample handling	For the determination of GM sulfate in pure, prepared and surface water samples	Microgram level (μg)	[[141], [142], [143]]
Microbiological Assays	Easy operation, minimal equipment required	Low sensitivity, prolonged experimental cycles, limited stability, high variability, and susceptibility to interference from impurities	Utilizes microbial inhibition to measure GM residue in samples.	Milligram (mg)	[127,147]
Immunochromatography (GICA)	Rapid and label-free, suitable for on-site testing	Insufficient sensitivity	GM detection based on antibody-antigen interaction using gold colloidal assays.	Microgram level (μg)	[147,148]
Surface Plasmon Resonance (SPR)	High specificity and sensitivity, no need for derivatization	Signal susceptibility to interference, low detection sensitivity, low technological maturity	Measures GM through real-time monitoring of antibody-antigen reactions.	Microgram level (μg)	[149]

### 5.1 GM detection methods in fermentation

The industrial production process of GM presents significant challenges for potency determination due to its structural complexity, multi-component nature, and the presence of various components and insoluble impurities in the culture medium. Traditional methods, such as agar diffusion [107], calculate titer by measuring the inhibition zone diameter against a test organism, yielding the total GM content but prone to human errors. Standard methods include pre-column derivatization liquid chromatography and liquid chromatography-tandem mass spectrometry. Colorimetric and UV spectrophotometric methods offer rapid and accurate titer determination during production or strain screening. Xu et al. established Levey's salt colorimetric method based on the insoluble complexes formed between GM amino groups and Levey's salt [108]. Chen et al. utilized GM's reaction with copper tartrate alkaline solution to form colored complexes measured at 560 nm, albeit with low sensitivity and accuracy [109]. UV spectrophotometry, widely cited in pharmacopeias, involves the Hantzsch reaction between GM amino groups and acetylacetone and formaldehyde in a borate-acetate buffer, albeit with challenges in maintaining consistent pH values [110]. Li et al. employed an *ortho*-phthalaldehyde (OPA) reagent for the ultraviolet determination of GM, yielding derivatives with UV absorption characteristics via nucleophilic addition Schiff base reaction [111]. For rapid screening of high-performance strains, Tian and Liu optimized a sodium phosphotungstate UV spectrophotometric method [61,112], while Zhu et al. introduced a novel high-throughput detection method using computer vision and machine learning [62]. Therefore, utilizing various GM detection methods in fermentation processes is critical for monitoring and optimizing production. These methods ensure the final product's quality and consistency and help identify potential bottlenecks and inefficiencies. This foundational understanding sets the stage for exploring GM detection methods in other fields, which will be discussed in the next section.

### 5.2 GM detection methods in other fields

In addition, a wide range of GM detection methods have been demonstrated and optimized across various fields (Table 2). Capillary electrophoresis (CE) emerged as an early and widely adopted detection method [[113], [114], [115], [116]]. Gukowsky et al. introduced a simple, rapid, and sensitive capillary electrophoresis colorimetric approach employing cysteamine-modified gold nanoparticles (cys-AuNPs) for detecting GM content in food [116]. Notably, this technique is more cost-effective, straightforward, and efficient than other industrial and commercial methods for antibiotic detection. It harbors significant potential as a swift screening technique for GM and analogous compounds in food and environmental samples. Fluorescence spectroscopy exploits the fluorescence emitted by certain substances in the excited state upon exposure to ultraviolet light, reflecting the substance's characteristics through excitation, collision, and emission processes, providing a qualitative or quantitative analysis method [117,118]. Gubernator et al. introduced an innovative method for GM determination in liposomes [118]. Under physiological pH conditions, the amino groups in GM molecules react with OPA, and the fluorescence of the product can be directly measured using a simple fluorometer. However, its limitation lies in avoiding or removing other molecules containing imino groups. Chemiluminescence (CL) is a form of molecular luminescence spectroscopic analysis, primarily grounded on the concept that the analyte concentration in the chemical detection system shows a linear quantitative relationship with the system's chemiluminescence intensity under specific conditions. It utilizes instrument-based detection of the chemical luminescence intensity to ascertain analyte content, serving as a trace analysis technique [[119], [120], [121], [122]]. Iranifam et al. developed a practical and sensitive flow injection chemiluminescence (CL) method for determining GM sulfate [120]. The study achieved high-throughput detection of 120 samples/h and effectively applied it to determine GM sulfate in formulations and water samples. Krzek et al. devised a derivative spectrophotometric technique to quantify GM levels in injectable solutions [121]. Furthermore, Fraihat introduced a new approach for GM analysis, utilizing the oxidation reaction of GM with permanganate under alkaline conditions, resulting in the formation of green manganate ions with peak absorption at 610 nm [122].

Due to its molecular structure lacking strong chromophores and high polarity, quantifying GM proves challenging with conventional high-performance liquid chromatography (HPLC) methods. Joseph et al. devised a Reverse Phase HPLC (RP-HPLC) approach using a pentafluorophenyl column and charged aerosol detector (CAD) to assess GM sulfate composition in ointments [123]. Furthermore, pre-column and post-column derivatization are effective techniques for improving aminoglycoside drug retention in reverse-phase chromatography, allowing for UV or fluorescence detection. Addressing the suboptimal separation of the C1 component, Lin et al. optimized various factors influencing GM C component and introduced formic acid instead of acetic acid in the mobile phase, enhancing separation efficiency and analysis time [124]. Stypulkowska et al. successfully employed a more common C_18_ column to establish a LC-CAD method for GM sulfate composition and related substances [125]. In addition, Patil et al. pioneered the application of HPLC with a Quality by Design (QbD) approach for detecting and separating GM sulfate in biodegradable implants [126]. Evaporative light scattering detection (ELSD) is a universal detection mode suitable for non-absorbing analytes [[127], [128], [129], [130], [131]].

Ultra-performance liquid chromatography-tandem mass spectrometry (UPLC-MS/MS) has garnered extensive applications across various domains, including pharmaceutical analysis, food analysis, and environmental assessment [[132], [133], [134], [135], [136]]. Inoue et al. formulated an efficient preparative technique grounded in High-speed counter-current chromatography/Mass spectrometry (HSCCC/MS) to purify GM C1a, C2, C2a, and C1 constituents from standard antibiotic powders, affirming the method's feasibility [135]. An et al. established a methodology for GM content determination in feed through solid-phase extraction coupled with liquid chromatography-tandem mass spectrometry, boasting detection and quantification thresholds of 0.03 mg/kg and 0.1 mg/kg, respectively, aligning with the detection standards for prohibited feed additives [136]. Colorimetric techniques have also been developed for GM determination [[137], [138], [139], [140]]. Li et al. introduced a novel method employing citrate-capped silver nanoparticles (AgNPs) as probes for GM determination [139]. It enables GM detection in human urine with a recovery rate of 97 %–103 %. Ul-Ain et al. described the green synthesis of gallic acid methyl ester-coupled silver nanoparticles and their application in colorimetric GM quantification [140]. The MG-AgNPs colorimetric probe used in this GM sensor offers stability, affordability, high sensitivity, and specificity, showing promise for on-site GM identification in actual samples like hospital waste. Recently, electrochemical methods have emerged for detecting GM [[141], [142], [143]].

Apart from the methods mentioned above, additional techniques for detecting GM residue encompass microbiological assays, immunochromatography, and surface plasmon resonance (SPR) technology. Microbiological assays exploit the ability to inhibit microbial growth for qualitative or quantitative analysis of residual GM in samples [127]. Although microbiological assays require minimal equipment and are straightforward to operate, they exhibit drawbacks, including low sensitivity, prolonged experimental cycles, limited stability, high variability, and susceptibility to interference from impurities. Ikram et al. synthesized epigallocatechin-3-gallate-coated silver nanoparticles (ECAgNPs) and employed them to fabricate an economical, sensitive, and highly selective GM sensor [144]. The interaction between epigallocatechin-3-gallate and silver was assessed using UV–visible and FT-IR spectroscopy, while dynamic light scattering (DLS), atomic force microscopy (AFM), and scanning electron microscopy (SEM) were utilized to examine the particle size and morphology. Anyakudo et al. introduced a rapid TLC-FID method for analyzing GM, etimicin, and amikacin in pharmaceuticals, employing balomycin as a quantitative internal standard to ensure expeditious analysis [145]. Chen et al. demonstrated the use of a microbial sensor based on MIL-53(Fe) for GM detection [146]. Yang et al. applied colloidal gold immunochromatographic assay (GICA) to detect GM residues in pork, with results showing excellent concordance with those obtained by enzyme-linked immunosorbent assay (ELISA), rendering it suitable for rapid on-site detection [147]. Zhou et al. quantified GM in milk using the check-III quantitative rapid detection analyzer with GICA, enabling simultaneous screening of samples within 20 min, a method suitable for large-scale screening at raw milk stations [148]. Wang et al. devised a rapid detection approach for GM residues in milk based on surface plasmon resonance (SPR) technology [149]. SPR technology offers advantages such as rapid detection, label-free analysis, and real-time monitoring, effectively overcoming the limitation of GM's lack of chromophores by combining with the high specificity and sensitivity of antibody-antigen reactions, thus enabling specific detection without derivatization. The method simplifies sample handling and mitigates the impact of derivatization on detection outcomes, making it particularly suitable for high-throughput detection of GM residues.

In summary, a wide range of GM detection methods have been developed and optimized across various fields, highlighting the growing need for sensitive, selective, and efficient analytical tools. Early methods like CE and fluorescence spectroscopy offered minimal derivatization but were somewhat limited in sensitivity and scope. More recent techniques, such as CL and HPLC, including RP-HPLC and HPLC-ELSD, provide greater accuracy and faster analysis, overcoming challenges associated with GM's high polarity and lack of solid chromophores. Advanced methods like UPLC-MS/MS stand out for their high sensitivity and versatility, finding applications in food safety and environmental monitoring. In addition, colorimetric and electrochemical techniques have gained traction as rapid, cost-effective alternatives for on-site testing samples such as dairy and water. Biotechnological approaches, including microbial detection, GICA, and SPR, offer high-throughput, user-friendly solutions, particularly in food and pharmaceutical safety contexts. In conclusion, these methods mark significant advancements in GM detection technology, consistently improving accuracy, speed, and application range to meet the diverse needs of various industries. As these technologies gain traction in diverse industries, a significant need arises for comprehensive market analysis to understand consumer demand, market trends, and the competitive landscape. This necessity will be addressed in the next section, focusing on the marketing analysis of GM.

## 6 Marketing analysis

The GM market is pivotal in global public health and disease management, emerging as a critical weapon against various bacterial infections amidst the escalating challenge of antibiotic resistance [32]. Multiple factors shape the growth trajectory of this market. Biosynthesis is a prevalent method for GM production, with fermentation primarily employing *Streptomyces* species, offering cost advantages albeit demanding stringent control measures to ensure product quality and safety. Yet, significant producers encounter constraints in augmenting GM production owing to limitations in strain performance and process technology [96]. GM is widely applied in both medical treatment and animal husbandry. In medical contexts, it is a crucial agent against bacterial infections encompassing respiratory tract, skin, and urinary tract infections. Similarly, animal husbandry plays a preventive and therapeutic role, bolstering animal health and the steady progress of husbandry practices [59]. Furthermore, the GM industry's evolution is propelled by diverse factors. The surge in antibiotic resistance urges pharmaceutical endeavors to explore novel antibiotic solutions. Concurrently, advancements in hospital care and clinical treatments escalate the demand for efficient antibiotics, thus fueling the GM market's expansion. Notably, escalating healthcare awareness progressively drives antibiotic demand, complemented by ongoing innovations in GM derivatives, further stimulating market growth. In addition, governmental support for healthcare and streamlined market access fosters a conducive environment for market growth [84]. Noteworthy statistics indicate that global sales of aminoglycoside antibiotics, including GM, reached $1.2–1.3 billion in 2016, with GM accounting for approximately $675 million. By 2022, global sales surged to about $900 million. Projections from commercial consulting entities anticipate a compound annual growth rate of 5.27 % for the global GM market, reaching $688 million by 2028, underscoring its dominance in the aminoglycoside antibiotic sector (source: https://www.globalmarketmonitor.com/). This projected growth is primarily spurred by sustained healthcare demand and the introduction of novel GM products. Geographically, the GM market spans North America, Europe, the Asia-Pacific, Latin America, the Middle East, and Africa, with developed countries exhibiting heightened demand due to advanced healthcare infrastructure and standards [119]. Conversely, developing nations witness a gradual rise in demand, buoyed by enhanced medical standards and infrastructure. Regulatory policies worldwide promoting judicious antibiotic use and curbing misuse significantly influence the GM market. Specific regulations, such as those governing the use of GM in assisted reproductive technologies and pediatric applications, underscore regulatory oversight. In addition, regulations regarding GM usage, dosages, and withdrawal periods in aquaculture in freshwater fish farming underscore evolving regulatory frameworks [131]. The World Health Organization's advocacy and guidance on antibiotic resistance have had a significant impact, driving continual improvements in production processes and product quality across the GM industry to address global medical challenges. Overall, a thorough marketing analysis of GM provides valuable insights into consumer preferences, market trends, and competitive dynamics. Understanding these factors is crucial for developing effective strategies that maximize market penetration and profitability.

## 7 Conclusion and prospects

GM, a broad-spectrum aminoglycoside antibiotic, is vital in treating Gram-negative bacterial infections. However, its efficacy is challenged by increasing bacterial resistance [13]. Notably, research on biosynthesis is crucial for discovering and developing novel aminoglycoside analogs with enhanced therapeutic properties. Glycosyltransferases are indispensable in the biosynthesis of aminoglycoside antibiotics, catalyzing the stepwise transfer of sugar moieties from primary metabolic donors to sugar acceptors, forming the core structure of these antibiotics. Specifically, glycosyltransferases in the kanamycin biosynthetic pathway exhibit broad substrate specificity, utilizing sugar donors derived from primary metabolism. Similarly, the post-modification system in GM biosynthesis demonstrates considerable potential for substrate flexibility. Therefore, the functional characteristics of glycosyltransferases and the post-modification system in GM biosynthesis can be harnessed through combinatorial biosynthesis approaches. These approaches enable the rational design and directed combination of distinct structural units, thus facilitating the development of novel aminoglycoside antibiotics. Furthermore, GM exhibits superior biological activity to kanamycin, suggesting that the additional modification groups in its structure may play a crucial role in enhancing its activity. Based on this observation, a recent study utilized synthetic biology and metabolic engineering techniques [150]. The researchers replaced functionally analogous components in GM biosynthesis with glycosyltransferases from the kanamycin biosynthetic pathway, engineering GM-producing strains to synthesize a novel trisaccharide intermediate with a substituted second sugar ring. The robust post-modification system in GM biosynthesis further modified this intermediate, generating a series of novel hybrid aminoglycosides, termed Genkamicins (comprising GM and kanamycin structural units). Metabolic engineering strategies were subsequently employed to direct the accumulation of specific target compounds, leading to the isolation of individual Genkamicin components. Genkamicin GK-C2a, in which the second substituted sugar ring was replaced with glucose instead of xylose, exhibited reduced ototoxicity in zebrafish compared to clinically used drugs. This finding suggests that Genkamicin GK-C2a, a single-component antibiotic, holds significant potential for future clinical applications and could be further developed as a safe and effective next-generation aminoglycoside antibiotic. This study presents a novel strategy for diversifying aminoglycoside antibiotic structures and offers new insights and approaches for developing next-generation aminoglycosides.

Overall, this review examines the advancements in GM production in *Micromonospora* over recent decades, covering biosynthetic mechanisms, metabolic engineering for high-yield strains, and fermentation optimization strategies. These studies offer theoretical insights and practical guidance for enhancing GM yield and quality. In addition, the review discusses detection methods for GM in various products, stressing the necessity of employing diverse advanced technologies for accurate quantification, given GM's broad application. Future research could leverage metabolic engineering and systems biology, including genome-scale metabolic models and gene expression analysis, to decipher the GM biosynthetic network, identify metabolic bottlenecks, and optimize production pathways. Moreover, novel high-throughput screening techniques could rapidly identify strain variations and fermentation conditions affecting GM production, expediting strain selection and optimization. With the rapid progress in sensing and information technologies, biosensors are increasingly applied in antibiotic fermentation, facilitating real-time detection of multiple parameters and enhancing our understanding of cellular metabolism. Furthermore, integrating multi-omics data and data science techniques, such as machine learning algorithms, can establish data-driven mechanistic models for precise fermentation control and optimization [62]. Regarding novel drug development, structural modification and functionalization can yield new GM derivatives with enhanced antibacterial activity, reduced side effects, and delayed resistance development [14,151]. These efforts to improve GM production will advance scientific methods for traditional antibiotic manufacturing. Delving into these research directions is expected to further enhance GM production efficiency and clinical application, offering more strategies and tools for combating resistant bacterial infections. However, attention must also be given to safety and environmental concerns in GM production, aiming for green and sustainable practices.

## CRediT authorship contribution statement

**Feng Xu:** Writing – original draft, Visualization, Resources, Investigation, Formal analysis, Data curation, Conceptualization. **Kaihao Hu:** Writing – original draft, Visualization, Data curation. **Ali Mohsin:** Writing – review & editing. **Jie Wu:** Resources, Data curation. **Lihuan Su:** Resources, Data curation. **Yuan Wang:** Resources, Data curation. **Rong Ben:** Resources, Data curation. **Hao Gao:** Resources, Data curation. **Xiwei Tian:** Writing – review & editing, Supervision, Project administration, Funding acquisition, Conceptualization. **Ju Chu:** Writing – review & editing, Supervision, Project administration, Conceptualization.

## Funding

This work was financially supported by the 10.13039/501100012166National Key Research and Development Program of China (2022YFC2105403), the Taishan Scholars Program, the Shanghai Pilot Program for Basic Research (22TQ1400100-14), the 10.13039/100007219Natural Science Foundation of Shanghai (23ZR1416500), the Frontiers Science Center for Materiobiology and Dynamic Chemistry (JKVJ1231036). Thanks for the financial support from the Arawana Charity Foundation.

## Declaration of competing interests

The authors declare that they have no known competing financial interests or personal relationships that could have appeared to influence the work reported in this paper.
